# Whole-Body Mechanics of Double-Leg Attack in Elite and Non-elite Male Freestyle Wrestlers

**DOI:** 10.3389/fspor.2020.00058

**Published:** 2020-06-10

**Authors:** Daichi Yamashita, Hiroshi Arakawa, Takahiro Wada, Kenichi Yumoto, Kotaro Fujiyama, Tomoyuki Nagami, Seshito Shimizu

**Affiliations:** ^1^Department of Sport Science, Japan Institute of Sport Sciences, Tokyo, Japan; ^2^Faculty of Physical Education, International Budo University, Katsuura, Japan; ^3^Department of Sport and Physical Education, Kokushikan University, Tokyo, Japan; ^4^Faculty of Sport Science, Nippon Sport Science University, Yokohama, Japan; ^5^Japan Wrestling Federation, Tokyo, Japan; ^6^College of Liberal Arts and Sciences, Kitasato University, Sagamihara, Japan; ^7^Sports Design Lab, Tokyo, Japan

**Keywords:** wrestling, kinematics, kinetics, olympic medalists, freestyle

## Abstract

This study examined the movement characteristics of the double-leg attack in elite and non-elite wrestlers. Twenty light-weight male wrestlers were divided by skill level: Elite group (*n* = 11) who participated in international-level competitions and Non-Elite group (*n* = 9) consisting of college-level wrestlers. Each wrestler performed the double-leg attacks against a defender. Three-dimensional coordinates of anatomical landmarks and the ground reaction force (GRF) of the trailing limb were analyzed. The forward velocity and displacement of the whole-body center of mass (COM) and the 7th cervical spine (C7), which represents the upper body, were calculated. Additionally, joint torques were calculated by a standard inverse dynamics method. No significant differences were observed between groups for movement duration and the C7 forward displacement, which relates to the interpersonal distance. Still, they were significantly correlated in the non-elite wrestlers, as well as in all wrestlers (*r* = 0.78, *p* < 0.05 and *r* = 0.65, *p* < 0.01, respectively). While there were no group differences in joint angles at both limbs and torques at the trailing limb, the time-to-peak resultant GRF was shorter, and peak resultant GRF was greater at the trailing limb in the elite wrestlers compared to that in the non-elite wrestlers (*p* < 0.05). There were no group differences in peak forward velocity of the COM and the C7. However, the C7 forward velocity at 0.20, 0.25, and 0.30 s and the C7 forward displacement at 0.35 s after the start of the attack was significantly greater in the elite wrestlers compared to that in the non-elite wrestlers (*p* < 0.05). This disparity in the C7 forward velocity made a positive contribution in forward displacement by 0.08 m at 0.35 s. Thus, during a double-leg attack, elite wrestlers quickly move their upper body forward while rapidly pushing-off the trailing foot reaching the defender's legs in advance of defensive actions, irrespective of the interpersonal distance. These characteristics may improve the success rate of the double-leg attack.

## Introduction

Wrestling is one of the oldest competitive sports with records of its practice since the ancient Olympic Games and it continues to be one of the most competitive sports in the world. Since its reintroduction to the Modern Olympic Games in 1896, two types of wrestling for men (freestyle and Greco-Roman) are practiced. The women's competition (freestyle) was introduced in 2004. While both styles share many similarities, Greco-Roman wrestling focuses only on attacks using the upper body, while freestyle wrestling involves the use of the entire body. A match consists of two 3-minrounds with a 30-s break, with the objective to put the opponent on their back, pin them (opponent's shoulders held against the mat for a while) or by scoring the highest number of points.

Wrestling is a high-intensity intermittent combat sport that requires substantial technical skills. A technical-tactical analysis of freestyle wrestling showed that leg attack moves (not permitted in Greco-Roman wrestling) were the most utilized technique for scoring points (Cipriano, 1993; Fujiyama et al., 2019). This rise in attack-oriented wrestling strategy (Tünnemann and Curby, 2016) could be due to the rule change, awarding wrestlers 2 or 4 points for a successful takedown instead of 1 point previously. Furthermore, winners in international wrestling competitions were also shown to complete almost twice as many successful leg-attack maneuvers than their opponents (Cipriano, 1993). At the 2015 world championship, the majority of the points scored by the gold medalist in each weight category were derived from the leg attack maneuvers (Tünnemann, 2016).

The effectiveness of this attack appears to be underpinned by the velocity of the movement and the short movement time. In rugby tackling, the purpose is similar to a leg attack in wrestling; stopping ball carrier's penetration and taking down the opponent. An attacker tries to make high-intensity collisions with an opponent (McLellan et al., 2011). In freestyle wrestling, the double-leg attack begins when the attacker takes a step toward the opponent with the leading foot from a staggered crouched position, lowering the body by bending the lower limbs while approaching the opponent. Once in position, the attacker drives their upper body into the opponent's abdomen and grabs the back of the knees while pulling the opponent to the ground (Mysnyk et al., 1994) (see Movie S1). The movement time of leg attack significantly correlated with the win-loss record in collegiate wrestlers (Whitley and Montano, 1992). These findings share similarity with the attack movements in Fencing, with higher peak forward center of mass (COM) velocity, horizontal peak ground reaction force (GRF), joint kinematics (range of motion), and joint kinetics (hip and knee torque) measured in more effective attacks that were completed by international level than collegiate level fencers (Guan et al., 2018).

While the double-leg attack could be more effective by reducing the distance between the attacker and opponent (decrease movement distance and duration), this would also inevitably increase the risk of an attack by the opponent. Therefore, the maintenance of a minimum engaged distance between players is key to executing both effective attack and defensive maneuvers. This was observed in kendo matches and play-tag games, where players attempt to maintain a distance from the opponent until the execution of either striking defensive movements (Kijima et al., 2012; Okumura et al., 2012). Presently, the kinematics and kinetics of double-leg attack in elite wrestlers remain uncertain, in which the success rate was almost double compared to non-elite wrestlers (Cipriano, 1993).

Therefore, the purpose of the present study was to evaluate the movement characteristics of the double-leg attack in elite wrestlers in comparison to non-elite wrestlers. In the present study, wrestlers performed double-leg attacks on a defender in their preferred distance, stance, and maneuvers as they would in a wrestling match. Our analysis focused on the movement characteristics of the upper, lower, and whole body, as well as the kinetics of the trailing limb.

## Materials and Methods

### Participants

Twenty light-weight (former 55-, 60-, or 66-kg) Japanese male wrestlers performed the double-leg attack against a defender. The elite group consisted of 11 wrestlers (age: 23.9 ± 4.0 y; height: 1.66 ± 0.03 m; mass: 67.5 ± 4.4 kg) who had participated in at least one United World Wrestling (UWW) competition from 2012 to 2015, which included three Olympic medalists. The non-elite group comprised of nine Japanese division-1 collegiate wrestlers (age: 19.6 ± 1.0 years; height: 1.69 ± 0.04 m; mass: 66.8 ± 3.4 kg) with no experience in UWW-registered international competitions. The sole defender was an international level wrestler (age: 28 years; height: 1.66 m, mass: 63 kg, former 60-kg class) who did not participate as an attacker in this study. This study was conducted according to the Declaration of Helsinki and was approved by the ethics committee of the Japan Institute of Sports Sciences (H26-047). All participants provided written informed consent.

### Procedures

The double-leg attack was recorded and analyzed using a 3-D optical motion capture system (200 Hz; Vicon-MX, Oxford, UK) consisting of 20 cameras and a force platform (1,000 Hz; 0.9 m × 0.6 m, type 9287B, Kistler, Winterthur, Switzerland). Before the experiment, the height of the defender's stance was determined during pilot testing. The three wrestlers in the elite group adopted their preferred staggered crouched stance, as they would in a wrestling match (one leg forward and the other leg back). Then, two markers were attached: the front side of the acromion and greater trochanter. The height of the markers were measured and normalized to each attacker's body height. The average normalized height of the markers at the acromion and great trochanter were 0.54 and 0.42, respectively.

**Figure 1:**
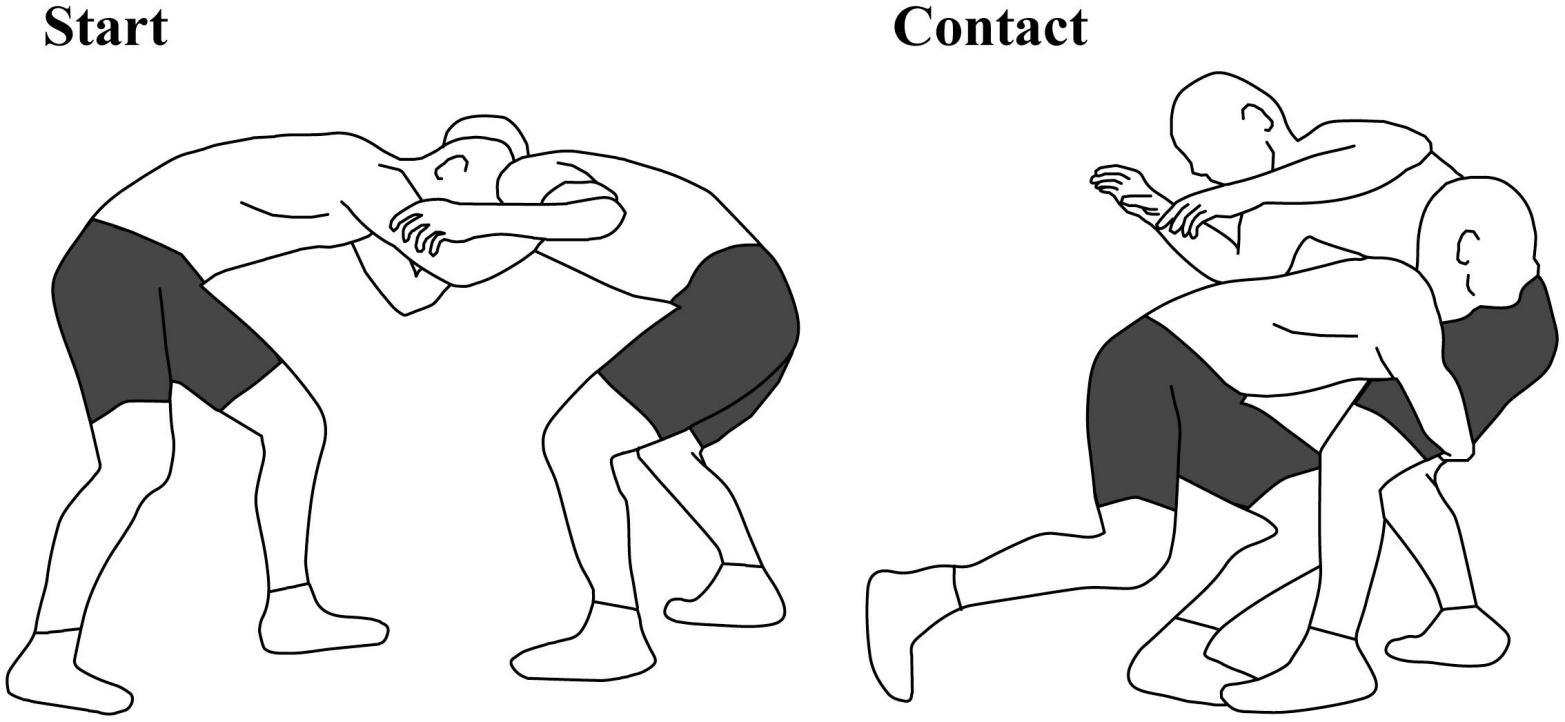
Schematic diagram of a double-leg attack from the starting position (left) to the ending position (right).

Before the start of each leg attack, both the attacker and the defender adopted their staggered position with hands contacting their shoulders or arms as they would in a wrestling match (see Figure 1). Then, the defender adjusted the foot position to mirror each attacker (either left or right foot forward) and held the same posture until the initiation for all double-leg attacks. The defender was asked to adjust their posture based on the attacker's height and the relative height of the front side of the acromion and greater trochanter to coincide closely with the pilot study. The height of the markers was strictly monitored in real time with Vicon Nexus software (v 1.8.5, Vicon Motion Systems, Oxford, UK). Preparatory movements (such as a small fluctuation movement and forward/back steps) preceding the attack were allowed to simulate actual match conditions. Following each trial, the attacker rated the quality of the attack on a scale from 1 (poor) to 5 (excellent). A minimum of four trials were completed in which two trials scored a rating of at least 4 or 5. Following motion analysis research for a throwing technique in judo (Ishii et al., 2017), we selected the highest-rated trial for further detailed analysis. When more than one trial scored the highest rating, the latest trial was analyzed.

### Data Collection

Forty-six reflective markers were placed on the attacker's body. Raw kinematic and GRF data were filtered using a fourth-order Butterworth digital low-pass filter with cut-off frequency of 20 Hz (Bisseling and Hof, 2006). To understand the whole body movement, the whole-body COM position was calculated as the weighted sum of a 14-segment model based on body-segment parameters (De Leva, 1996). The GRF at the trailing limb was recorded. All GRF data were normalized by body weight, with the addition of 0.25 kg the foot to account for the mass of the wrestling shoe. The origin of the global axes was set to the corner of the force platform, and the vertical axis was set to the upward direction.

The pelvis, thigh, shank, and foot anatomical coordinate systems were established the same as those used by Inaba et al. (2013). They were all right-handed orthogonal systems determined using the cross products of unit vectors defined by anatomical landmarks on each segment. The three-dimensional kinematics was computed using a Cardan sequence (x–y′-z″) (Winter, 2009). Three-dimensional net joint torques were calculated by the standard inverse-dynamics calculation, using GRF data and kinematic data. Joint torques were normalized to the body mass of each participant.

Attacks targeting the legs are typically initiated by extending the arms, flexing the hip, and/or bending the knees; in order to lower the attacker's COM and to reach for the opponent's legs (Mysnyk et al., 1994). Therefore, the start of the double-leg attack begun at the moment vertical velocity of the COM exceeded −0.1 m/s (negative to represent downward velocity). Previous research on Taekwondo kicking and rugby tackling defined the end of the attack as the moment of contact (Cheng et al., 2015; Kawasaki et al., 2018) ADDIN EN.CITE. We defined the timing when the horizontal acceleration of the defender's contralateral greater trochanter marker exceeded 10 m/s^2^ because an ipsilateral marker was often hidden by the attacker. Acceleration is estimated by double-differentiation of the position of the marker.

**Figure 2:**
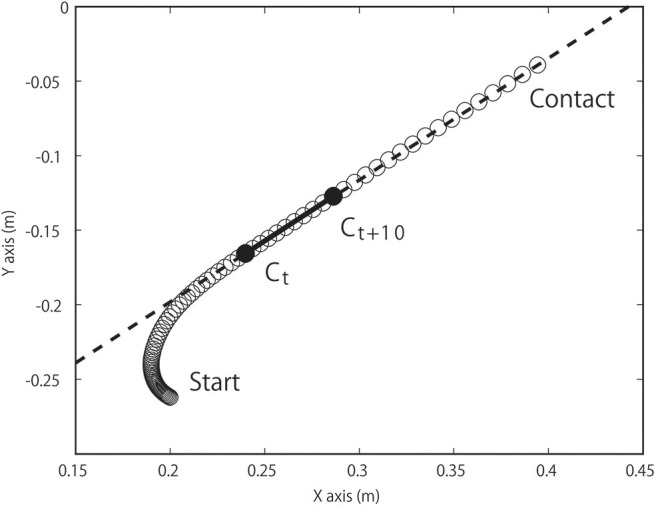
The COM trajectory was used to define the fore-aft axis in the horizontal plane of one trial. The fore-aft axis (dotted line) was along the line connecting the COM coordinates when the COM horizontal speed exceeded 1 m/s (C_*t*_) with that 10 frames later (0.05 s later, C_*t*+10_). X and Y denote global coordinates. COM, center of mass.

A local coordinate system was defined to enable the quantification of the approaching behavior of the attacker to the defender. Axis alignment was determined using the COM due to its almost linear trajectory in the horizontal plane during approaches (Figure 2). The fore-aft axis was along the line connecting the COM coordinates when the COM horizontal speed exceeded 1 m/s (C_*t*_) with that in 10 frames later (0.05 s later, C_*t*+10_). The mediolateral axis was defined as the cross product of the fore-aft axis and the vertical axis. We confirmed that the maximum mediolateral displacement during each wrestler's trial was very small (mean ± SD; 0.041 ± 0.011 m). The kinematic and GRF data were rotated to align with the local coordinate system using a rotation matrix (Movie S2).

In this study, the 7th cervical spine (C7) marker was considered to represent the upper body for two reasons: (1) the C7 forward movement is essential to reach a defender's legs because the typical and ideal contact point of an attacker is between the shoulder and neck (Mysnyk et al., 1994), and (2) the markers around that point (i.e., a sternoclavicular joint and shoulder) were often hidden and unable to track due to the contact. To identify movement characteristics in the vertical axis, the initial and final height of the C7, COM, and hip were calculated. The COM and hip were considered to represent the whole and lower body, respectively. The hip coordinate was defined as the midpoint between the right and left hip joint center, which was estimated by a previous study (Kurabayashi et al., 2003).

As indices of the quickness of the movement, the entire movement duration (from start to contact) and GRFs were calculated, as well as the COM and C7 forward and vertical velocities and displacements. Finally, the C7 forward displacement relates to the interpersonal distance because the attacker drives his upper body (near the C7 marker) into the contact point (opponent's abdomen). All numerical calculations were performed using the MATLAB 2011a (The MathWorks, Inc., MA, USA).

### Statistical Analysis

Data are reported as means and standard deviation. All statistical analyses were performed using IBM SPSS Statistics 19.0 (IBM Inc., Tokyo, Japan). The Kolmogorov-Smirnov test was employed to assess the normality of the data distribution. The F tests were used to determine whether these variances between the two groups were equal. When the variances between the two groups were equal, unpaired *t*-tests were performed to evaluate group differences. Pearson's correlation coefficient was used to evaluate the relationship between movement duration and interpersonal distance. As the shortest duration of movement among the 20 wrestlers was 0.365 s, two-way repeated-measures ANOVAs were performed to assess group differences over time (0.05, 0.10, 0.15, 0.20, 0.25, 0.30, and 0.35 s) in terms of the joint angle, joint torque, the C7 and COM forward velocity, and displacement. *Post hoc* analyses were performed using pairwise comparisons with Bonferroni correction. Greenhouse-Geisser adjustments were used to correct for violations of sphericity, when appropriate. Cohen's *d* and partial eta-squared values ($\eta_{p}^{2}$) were reported as a measure of effect size. Significance was set at *p* < 0.05 for all statistical analyses.

## Results

We first evaluated the attacker's posture at the beginning and end of the double-leg attack (Table 1). There were no group differences in the C7, COM, or hip height at the beginning of the movement. However, the COM and hip height of the attackers at the moment of contact were higher in the elite group compared to that in the non-elite group (*d* = 1.23, *p* = 0.013; *d* = 1.12, *p* = 0.021, respectively).

**Table 1:** Initial and final posture of the double-leg attack in the elite and non-elite groups (mean ± SD).

	**Elite**	**Non-elite**	**Cohen's *d***	***p*-value**
**INITIAL POSTURE**				
C7 height (m)	1.02 ± 0.06	1.02 ± 0.05	0.12	0.786
COM height (m)	0.72 ± 0.03	0.72 ± 0.03	0.23	0.610
Hip height (m)	0.74 ± 0.02	0.75 ± 0.03	0.14	0.764
**FINAL POSTURE**				
C7 height (m)	0.84 ± 0.03	0.80 ± 0.06	0.89	0.064
COM height (m)	0.55 ± 0.05	0.48 ± 0.06^*^	1.23	0.013
Hip height (m)	0.53 ± 0.13	0.41 ± 0.10^*^	1.12	0.021

*COM, center of mass; C7, seventh cervical spine*.

* *Significant difference between groups (p < 0.05)*.

**Figure 3:**
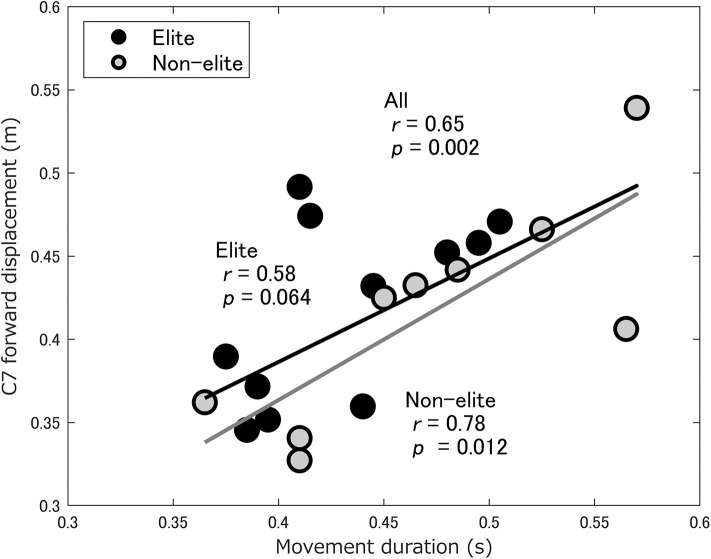
Scatterplot of the movement duration against the C7 forward displacement in the elite (black circles) and non-elite (gray circles) wrestlers. The black line indicates the correlation between the variables among all wrestlers. The gray line indicates the correlation between the variables in non-elite wrestlers.

There were also no group differences in movement duration, the C7 and COM forward displacement, or peak forward velocities of the C7 and COM (Table 2), but movement duration was significantly correlated with the C7 forward displacement in the non-elite wrestlers, as well as in all wrestlers (*r* = 0.78, *p* = 0.012; *r* = 0.65, *p* = 0.002, respectively) (Figure 3).

**Table 2:** Movement duration and forward movement characteristics of the double-leg attack in the elite and non-elite groups (mean ± SD).

	**Elite**	**Non-elite**	**Cohen's *d***	***p* value**
Movement duration (s)	0.43 ± 0.05	0.47 ± 0.07	0.70	0.136
C7 forward displacement (m)	0.42 ± 0.06	0.42 ± 0.07	0.04	0.934
COM forward displacement (m)	0.38 ± 0.06	0.41 ± 0.06	0.53	0.253
C7 peak forward velocity (m/s)	2.50 ± 0.23	2.58 ± 0.21	0.37	0.420
COM peak forward velocity (m/s)	2.28 ± 0.18	2.42 ± 0.16	0.77	0.106

*COM, center of mass; C7, seventh cervical spine*.

* *Significant difference between groups (p < 0.05)*.

**Figure 4:**
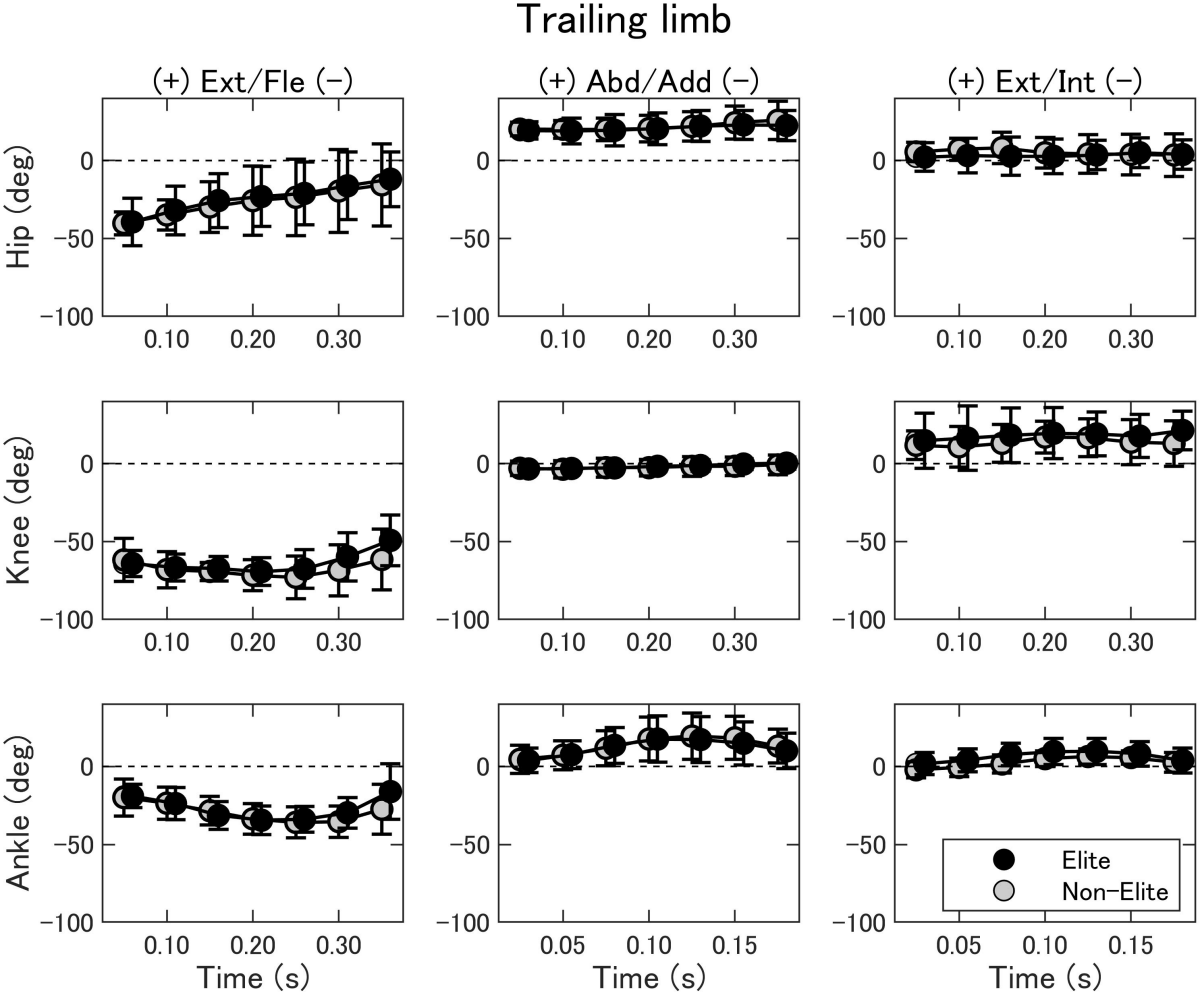
Joint angles of the hip (upper), knee (middle), and ankle (bottom) of the trailing limb during the double-leg attack in the elite (black) and non-elite (gray) groups. Ext/Fle, extension/flexion; Abd/Add, abduction/adduction; Ext/Int, external/internal rotation.

The joint kinematics of the trailing limb are summarized in Figure 4. The two-way ANOVA showed no interaction nor main effect of the group (*p* > 0.05). In general, the hip joint extended throughout the movement; on the other hand, the knee and ankle joint flexed at the beginning and extended before the contact. The changes in joint movements in the frontal and transverse planes were small.

**Figure 5:**
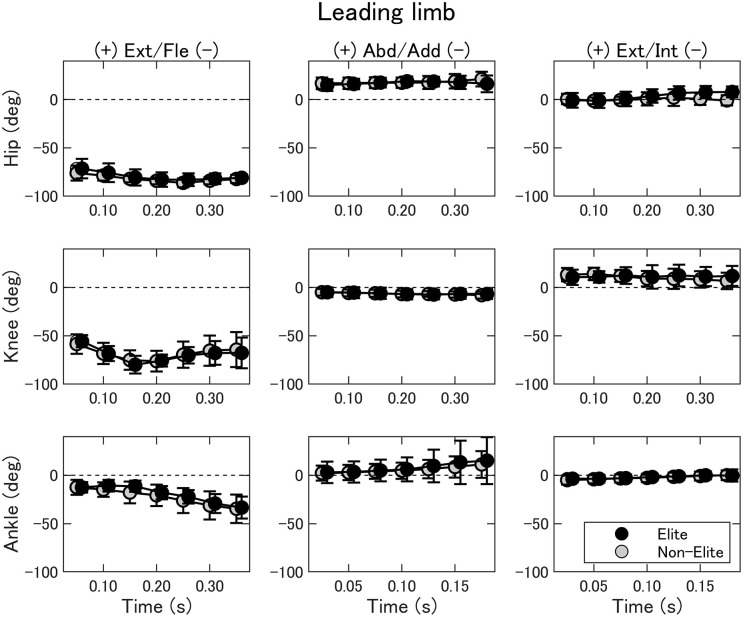
Joint angles of the hip (upper), knee (middle), and ankle (bottom) of the leading limb during the double-leg attack in the elite (black) and non-elite (gray) groups. Ext/Fle, extension/flexion; Abd/Add, abduction/adduction; Ext/Int, external/internal rotation.

The joint kinematics of the leading limb are summarized in Figure 5. The two-way ANOVA showed no interaction nor main effect of the group (*p* > 0.05). In general, the hip and knee joint flexed at the beginning; on the other hand, the ankle joint dorsiflexed before the contact. The changes in joint movements in the frontal and transverse planes were small.

**Figure 6:**
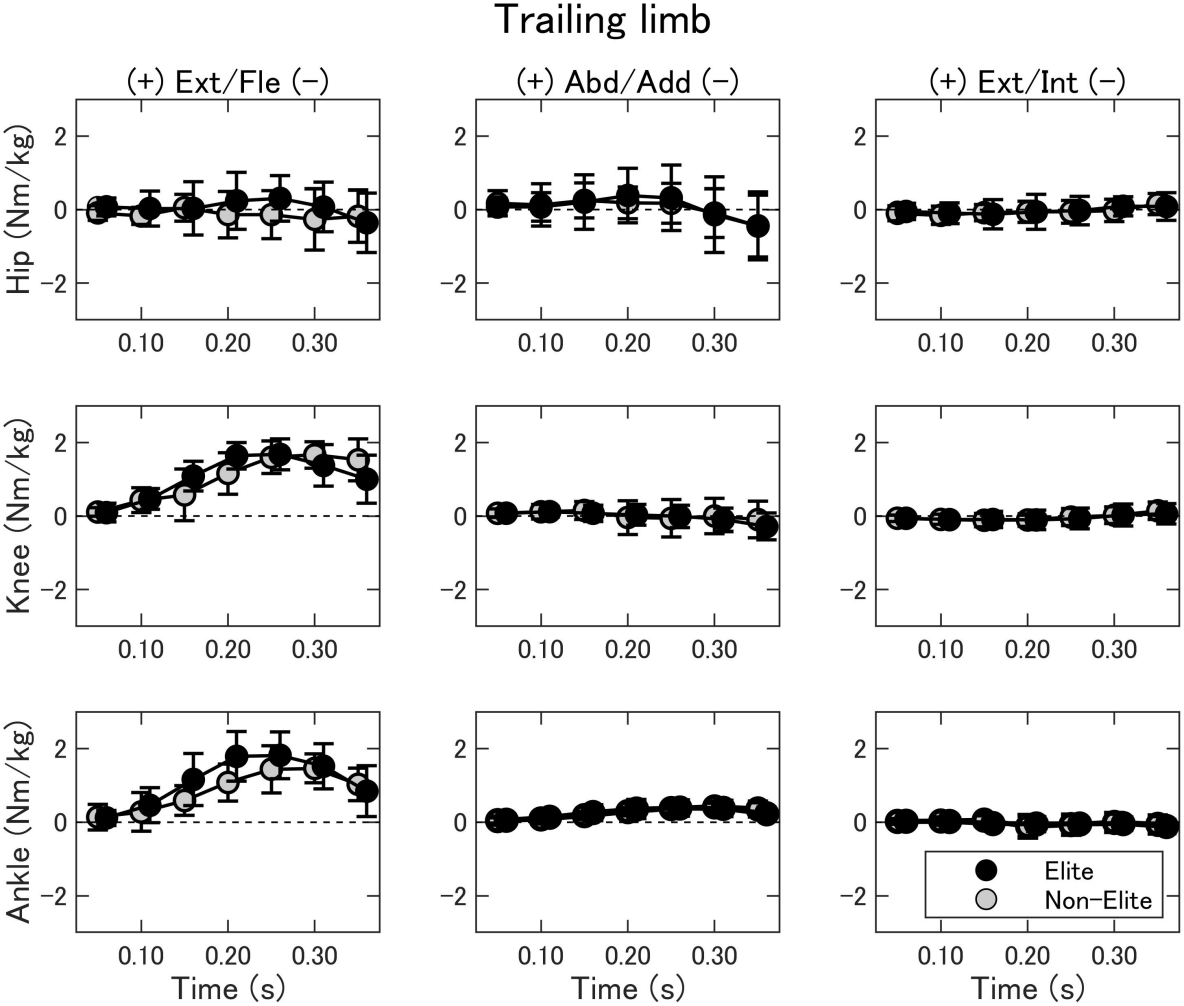
Joint torques of the hip (upper), knee (middle), and ankle (bottom) of the trailing limb during the double-leg attack in the elite (black) and non-elite (gray) groups. Ext/Fle, extension/flexion; Abd/Add, abduction/adduction; Ext/Int, external/internal rotation.

The joint torques of the trailing limb are summarized in Figure 6. The two-way ANOVA showed no interaction nor main effect of the group (*p* > 0.05). In general, the knee and ankle joint mainly produced extension torque. The changes in joint movements in the frontal and transverse planes were small, except the hip adduction.

There were group differences in the peak vertical and resultant GRFs (Table 3). Although the time-to-peak forward and resultant GRFs were significantly shorter in the elite wrestlers compared to that in the non-elite wrestlers (*p* < 0.05), there were no group differences in the time-to-peak vertical GRF.

**Table 3:** GRFs during the double-leg attack in the elite and non-elite groups (mean ± SD).

	**Elite**	**Non-elite**	**Cohen's *d***	***p*-value**
Peak vertical GRF (N/BW)	1.27 ± 0.24	1.04 ± 0.16^*^	1.09	0.026
Time-to-peak vertical GRF (s)	0.24 ± 0.05	0.27 ± 0.04	0.58	0.210
Peak forward GRF (N/BW)	0.99 ± 0.17	0.88 ± 0.19	0.61	0.19
Time-to-peak forward GRF (s)	0.26 ± 0.06	0.34 ± 0.07^*^	1.19	0.016
Peak resultant GRF (N/BW)	1.60 ± 0.28	1.34 ± 0.25^*^	0.98	0.042
Time-to-peak resultant GRF (s)	0.25 ± 0.05	0.30 ± 0.05^*^	0.96	0.046

*GRF, ground reaction force; BW, body weight. The GRFs were normalized by body weight*.

* *Significant difference between groups (p < 0.05)*.

**Figure 7:**
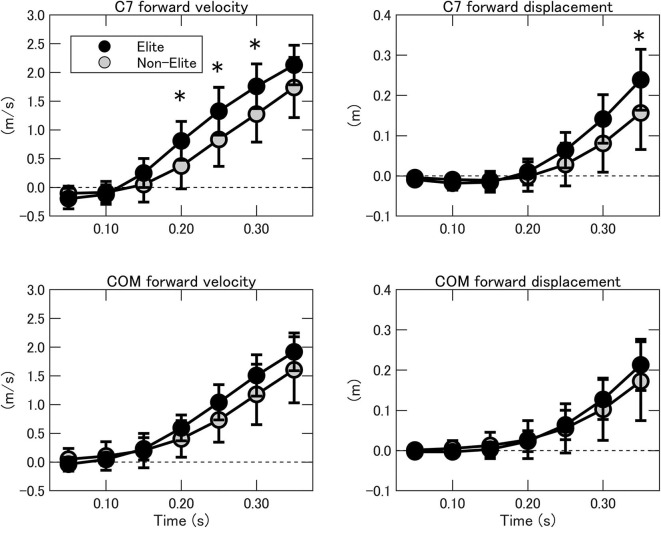
The C7 (top) and COM (bottom) forward velocities during the double-leg attack in the elite (black) and non-elite (gray) groups. COM, center of mass; C7, seventh cervical spine. *Significant difference between groups (*p* < 0.05).

For the fore-aft axis, significant interactions between group and time were found for the C7 forward velocity [*F*_(1.886, 39.953)_ = 4.889, *p* < 0.05, $\eta_{p}^{2}$ = 0214], and the C7 forward displacement [*F*_(1.140, 20.521)_ = 5.926, *p* < 0.05, $\eta_{p}^{2}$ = 0248] (Figure 7), but not found for the COM forward velocity [*F*_(1.411, 25.395)_ = 3.551, *p* = 0.058, $\eta_{p}^{2}$ = 0165] and the COM forward displacement [*F*_(1.177, 21.182)_ = 2.131, *p* = 0.157, $\eta_{p}^{2}$ = 0106]. The C7 forward velocity at 0.20, 0.25, and 0.30 s and forward displacement at 0.35 s showed significantly greater value in the elite wrestlers compared to that in the non-elite wrestlers (*p* < 0.05).

## Discussion

The purpose of the present study was to clarify the movement characteristics of the double-leg attack in elite wrestlers. No differences were observed for joint kinematics and kinetics between elite and non-elite wrestlers. Also, no group differences were observed for movement duration, the C7 and COM peak forward velocity, and peak forward GRF. However, the forward velocity of the C7 at 0.20, 0.25, and 0.30 s and the forward displacement at 0.35 following the start of the double-leg attack were found to be significantly greater in elite wrestlers compared to the non-elite wrestlers. These findings seem to be trivial but novel because there were some group differences in even the most basic leg-attack technique (attack a static opponent), which is explained in a drill book (Welker, 2012). Previous researches focused on the other technical aspects of elite wrestlers, such as balance braking action before leg attacks (Ito et al., 2019) and technical diversity (López-González, 2015).

Most of the joint motions and torque production were performed in the sagittal plane. For the lower-limb joint kinematics and kinetics, there were no group differences between elite and non-elite wrestlers. The outcome that the COM propulsive velocity was not different between groups is in contrast to previous studies on lunge motion by athletes of different levels. For instance, Guan et al. (2018) found that international level fencers performed greater hip and knee torque than intermediate fencers, and peak knee torque was correlated with the COM propulsive velocity.

A previous study reported that collegiate wrestlers with a shorter movement time using the double-leg attack had a higher win-loss percentage during matches (Whitley and Montano, 1992), which is inconsistent with the lack of group differences in movement duration in the present study. This might be due to the different competency levels of the participants in either study. Though collegiate level wrestlers constituted the non-elite group in the present study, these wrestlers still belonged to three of the top wrestling universities in Japan that were ranked in the top five positions for the eastern part of Japan in 2014. The movement duration cannot be directly compared between the studies because of differences in definitions; however, the inter-participant coefficient of variation in the previous study was 30.7% (mean, 0.40; SD, 0.12 s), which is much higher compared to that in the current study (elite group: coefficient of variation, 10.7%; mean, 0.43; SD, 0.05 s; non-elite group: 15.1%; mean, 0.47; SD, 0.07 s) (Table 3). Moreover, the results of the present study are consistent with those of a study examining movement duration in a judo-specific throwing technique “seoi-nage” in international-level and collegiate-level judokas (Ishii et al., 2017). These studies suggested that aspects other than movement duration are essential when approaching an opponent in a combat sport at a high level.

In the present study, the movement duration of a double-leg attack was correlated with the forward displacement of the upper body in the non-elite wrestlers, as well as in all wrestlers (Figure 3). Although a reduced interpersonal distance can be beneficial in terms of reaching the opponent's legs faster, there is also a greater risk of one's legs being reached by the opponent. Okumura et al. (2012) showed that kendo players maintain a preferred interpersonal distance between each other to balance between the gain and loss of offensive and defensive actions. This suggests that movement duration depends on the preferred interpersonal distance in the initial conditions, rather than skill level, and that movement duration is not necessarily an important factor for elite wrestlers.

The present results suggest that it is more important to move the upper body fast during the approach (0.20–0.30 s), rather than at the contact point. This difference in forward velocity made a difference in forward displacement by 0.08 m at 0.35 s. In a match, a defender blocks an attack to their legs by stepping backward and holding the attacker's upper body. Even when the defender's legs are grabbed, he holds the attacker's upper body and sprawls their legs backward, preventing the attacker from getting control of their body while they attempt counter-offensive actions. As a result, the success rate of leg attacks in winners and losers is 74% and 40% at international tournaments (Cipriano, 1993) and 100 and 66.7% at a Japanese tournament (Fujiyama et al., 2019), respectively; even winners are not always successful. Therefore, there appears to be a minimum requirement to approach a distance that allows one to reach a defender's legs in advance of the defender taking defensive actions, irrespective of the interpersonal distance.

In this study, the time-to-peak resultant GRF was shorter, and peak resultant GRF was greater at the trailing limb in the elite wrestlers compared to that in the non-elite wrestlers (Table 3). Typically, upper body propulsion is decomposed into the COM propulsion and that derived from its rotation about the COM. When the GRF vector does not run through the COM, the upper body rotates around the COM because of a moment produced around the COM. Therefore, the present results also suggest that a faster and greater push-off at the trailing limb helps to move the upper body faster relative to the COM. In this study, we could not get GRF data from the leading limb because wrestlers usually step forward between the opponent's legs (Mysnyk et al., 1994). Therefore, it is still unknown whether the leading limb might produce different force production patterns between groups.

Moreover, once a double-leg attack has been initiated, it can be finished in numerous ways to attempt takedown (Mysnyk et al., 1994). Physically, the production of enough resultant applied moment about the opponent's body is needed to perform a takedown. There are two ways to achieve this: by applying a forward force to their upper body and/or by applying a backward force to their lower body. In the present study, there were no group differences in the COM and C7 forward velocities on contact (Figure 7). This suggests that it is important to catch an opponent's lower legs and pull them, with contact around an opponent's waist. Indeed, success may depend not only on technical aspects but also on physical aspects. For example, Horswill et al. (1989) reported that successful wrestlers presented with higher anaerobic peak power in the upper limbs. Also, handgrip strength changes in wrestlers during the developmental years because of sport-specific training adaptations (Gerodimos et al., 2013). These physical characteristics of the upper limbs appear to be beneficial for catching and pulling the opponent's legs.

Relative to that in the non-elite wrestlers, elite wrestlers performed the double-leg attack with a smaller COM downward movement, caused by a smaller hip downward movement. Also, elite wrestlers produced greater peak vertical GRF. One possible advantage of this may be that the movement pattern of elite wrestlers is more robust to the external force applied by the opponent. Wrestlers slip or lose their balance when the horizontal GRF applied to the feet by the opponent exceeds the frictional force, which is determined by the vertical GRF and the coefficient of friction for the feet on the ground (Ekstrand and Nigg, 1989). A large downward displacement of the COM results in the small vertical force applied to the feet, which may lead to slip or lose their balance.

In conclusion, the current study revealed the movement characteristics of the double-leg attack in elite and non-elite wrestlers. Elite wrestlers move their upper body quickly, while quickly pushing-off the trailing limb. Of course, it is also essential for wrestlers to execute other techniques besides the double-leg attack to score points. International-level wrestlers have their winning strategies to disrupt the opponent's balance (Ito et al., 2019) and use their preferred techniques, such as single-leg attacks, counters, and throws (Tünnemann, 2016). However, our research found some kinetic and kinematic differences between skill levels, even in the most basic leg-attack technique. These results re-emphasize the importance of this basic movement, which coaches should take into account. Further study is needed to clarify the characteristics of other wrestling-specific techniques in elite wrestlers in a realistic wrestling match.

## Data Availability Statement

The datasets generated for this study are available on request to the corresponding author.

## Ethics Statement

The studies involving human participants were reviewed and approved by Japan Institute of Sports Sciences. The patients/participants provided their written informed consent to participate in this study.

## Author Contributions

DY and HA worked on study design, data collection, data analysis, and manuscript preparation. TW and KY worked on study design and data collection. KF worked on study design data collection. TN and SS worked on study design, data collection, and manuscript preparation.

## Conflict of Interest

The authors declare that the research was conducted in the absence of any commercial or financial relationships that could be construed as a potential conflict of interest.

## Supplementary Material

Supplementary Video 1 A 3-D view of the typical sequence of movement for the double-leg attack. The red arrow represents the ground reaction force, and the red circle shows the center of mass.

Supplementary Video 2 A 2-D view of the typical sequence of movement for the double-leg attack. The red arrow represents the ground reaction force, and the red circle shows the center of mass.
